# Choroidal atrophy and loss of choriocapillaris in convalescent stage of Vogt-Koyanagi-Harada disease: *in vivo* documentation

**DOI:** 10.1186/1869-5760-4-9

**Published:** 2014-03-22

**Authors:** Hossein Nazari, Amirhossein Hariri, Zhihong Hu, Yanwei Ouyang, SiriniVas Sadda, Narsing A Rao

**Affiliations:** 1 Department of Ophthalmology, Keck School of Medicine of the University of Southern California, Los Angeles, CA 90033, USA; 2 Doheny Eye Institute, Los Angeles, CA 90033, USA; 3 USC Eye Institute, University of Southern California, 1450 San Pablo Street, DVRC211, Los Angeles, CA 90033, USA

**Keywords:** Vogt-Koyanagi-Harada disease, Choriocapillaris, Choroidal vessels

## Abstract

**Background:**

The aim of this study was to determine the clinical significance of posterior choroidal thickness and vascular changes in the convalescent stage of Vogt-Koyanagi-Harada disease (VKH). Macular spectral domain optical coherence tomography (SD-OCT) images of 22 eyes of 13 consecutive patients with VKH at the convalescent stage were compared to 17 eyes of 9 age/sex/refraction-matched normal subjects. The choriocapillaris layer, medium choroidal vessels (Sattler's layer), and large choroidal vessels (Haller's layer) were assessed in foveal SD-OCT scans. The presence and the extent of disruption of outer retinal structures were also noted. Inner and outer choroid boundaries were manually drawn on horizontal raster SD-OCT scans, and choroidal thickness and volume maps were generated. Correlation analysis was run to assess the association of the above parameters in the VKH patients compared to the normal subjects.

**Results:**

In the eyes with convalescent stage of VKH, mean choroidal thickness in the foveal central subfield (200 ± 60 μm) was lower than in matched controls (288 ± 40 μm) (*P* < 0.0001). A thinner sub-macular choroid correlated with a lower visual acuity in uveitis eyes (Pearson correlation, *r* = -0.5089, *P* = 0.005). While the choriocapillaris layer was continuous and intact in all control eyes, various degrees of choriocapillaris loss were observed in 11 eyes (50%) with VKH (*P* < 0.0001). In these patients, the presence of outer retinal disruption was associated with a lower visual acuity (Spearman correlation, *P* < 0.001).

**Conclusions:**

The choroid is significantly thinner and the choriocapillaris layer is disrupted in the eyes with convalescent stage of VKH. Evaluation of the choriocapillaris in SD-OCT scans may be a useful surrogate marker for visual function in the convalescent stage of VKH.

## Background

Vogt-Koyanagi-Harada disease (VKH) is a bilateral posterior uveitis or panuveitis that typically presents with distinct clinical features based on the duration and stage of the disease [1,2]. The acute stage of VKH is followed by the convalescent stage of the disease, and the latter is characterized by resolution of retinal detachments with disappearance of cells in the anterior chamber and vitreous with characteristic sunset glow fundus changes. Usually, such convalescent stage develops 12 weeks after the onset of VKH. Histopathologic studies of acute VKH reveal diffuse, uveal infiltrations of mononuclear inflammatory cells, primarily lymphocytes, interrupted by focal aggregates of epithelioid histiocytes and multinucleated giant cells, with sparing of the choriocapillaris from the inflammatory infiltration. The convalescent stage of the disease reveals loss of choroidal melanocytes with focal infiltration of few lymphocytes [3,4]. The patients with long-standing duration of the convalescent stage may show choroidal atrophy and loss of choroidal melanocytes [3,5,6]. Our knowledge of choroidal vascular changes in VKH is limited in part due to inherent limitations of imaging studies such as angiography to evaluate intervascular choroidal stromal changes [6].

Until recently, ophthalmic ultrasound, indocyanine green angiography, and histology have been the only modalities to study choroidal stroma. In recent years, spectral domain optical coherence tomography (SD-OCT) has been used to evaluate both the normal choroid and the choroid in inflammatory and degenerative diseases [7-9]. Previously unseen details of the choroidal stroma and vasculature, including the choriocapillaris layer and the diameter and distribution of medium (Sattler's layer) and large (Haller's layer) choroidal vessels, are imaged in SD-OCT scans [10,11]. It is suggested that further insight into choroidal vascular morphology using SD-OCT imaging could potentially improve diagnosis and management of patients with ocular disease affecting the choroid [10,11]. A few attempts have been made to analyze choroidal vessels by manual and automated segmentation of SD-OCT scans in macular degeneration and in normal eyes [11-13]. Nevertheless, SD-OCT evaluations of choroidal vasculature in ocular inflammatory diseases in which the choroid is the major site of involvement are scarce.

SD-OCT-assisted monitoring of choroidal thickness, volume, and microstructure is considered helpful in estimating the burden of inflammation in the choroid and predicting the potential for recovery in acute VKH [14-17]. However, information regarding the SD-OCT features of the choroid in non-acute-phase VKH is limited [4,14-18]. Here, we used a qualitative approach to evaluate sub-foveal choroidal vasculature and choroidal thickness in SD-OCT scans to determine the clinical significance of the choroidal vascular changes and thickness in VKH during the convalescent stage.

## Methods

### Subjects

We retrospectively collected data from 26 eyes of 13 consecutive patients with VKH at the convalescent stage [19] and 17 normal eyes from 9 healthy individuals who were seen between 2010 and 2012. The normal controls were from a pool of volunteers with no evidence of eye disease.

All subjects underwent a complete ophthalmic examination and SD-OCT imaging. All control subjects had a best-corrected visual acuity (BCVA) of 20/20 or better and normal eye examination findings. All consecutive patients with the diagnosis of VKH based on the revised diagnostic criteria for VKH [20] were included if they were followed for at least 3 months after initial presentation with acute VKH, showed resolution of retinal detachment and cells in the anterior chamber and vitreous, and displayed sunset glow fundus changes [4]. Clinical information including demographic data, medical and ocular history, BCVA, slit-lamp biomicroscopy and fundus examination findings, and treatment details were collected from the medical records. The study was approved by the Institutional Review Board of the University of Southern California and conformed to the tenets set forth in the Declaration of Helsinki.

### Scanning protocol and development of choroidal thickness and volume maps

**Figure 1:**
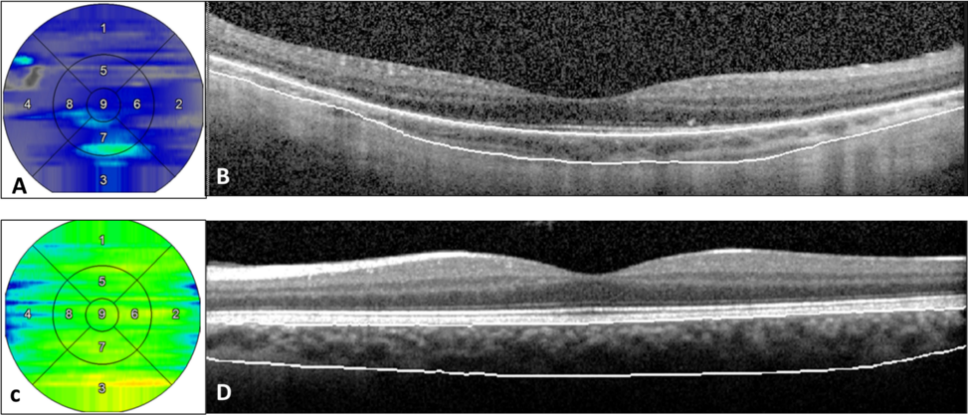
**Choroidal thickness maps and spectral domain optical coherence tomography scans.** Choroidal thickness maps in a patient with Vogt-Koyanagi-Harada disease at the convalescent stage **(A)** and in a normal control **(C)**. Respective spectral domain optical coherence tomography scans passing through the fovea with marking of choroidal boundaries are presented in panels **(B)** and **(D)**.

The SD-OCT scans were obtained with the Spectralis HRA + OCT (Heidelberg Engineering, Heidelberg, Germany). SD-OCT data were exported and imported into Doheny Image Reading Center OCT grading software (3D-OCTOR) as described in detail before [21,22]. The inner and outer boundaries of the choroid were manually drawn on all B-scans to allow computation of thickness and volume values for the entire scanned region. The choroid was defined as the outer boundary of the retinal pigment epithelium (RPE)-Bruch's membrane complex to the inner boundary of the hyperreflective sclera (Figure 1). All segmentations were performed by two independent, masked graders. Interobserver variability in choroidal thickness measurement was compared using Bland-Altman plots. Reproducibility was also assessed using the intraclass correlation coefficient.

Choroidal thickness and volume values were reported using a modified macular Early Treatment Diabetic Retinopathy Study (ETDRS) grid layout to facilitate analysis and comparison. ETDRS subfields consist of a central circle with a diameter of 1,000 μm that is surrounded by two larger circles, all centered on the fovea. The middle and outer circles encompass an area with a diameter of 3,000 and 5,000 μm, respectively. The sub-foveal choroid was defined as the choroid included within the central circle. The parafoveal choroid was defined as the choroid contained within the inner circular band with a width of 1,000 μm circling the foveal area. The entire posterior choroid was defined as the portion of the choroid within the 5,000-μm-diameter circle centered on the fovea.

### Qualitative evaluation of the choroidal vessels and retina

**Figure 2:**
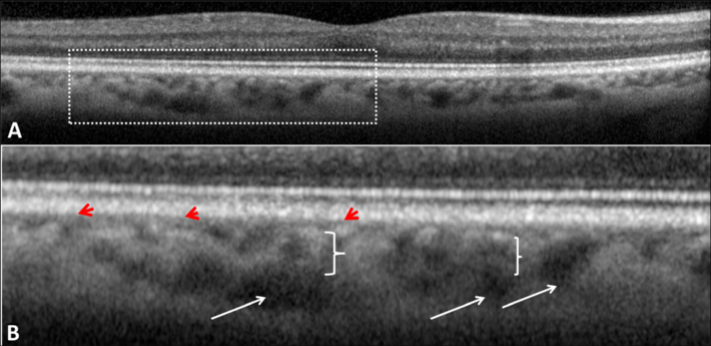
**Choroidal vasculature in a normal eye and its enlarged view.** Choroidal vasculature in a normal eye as revealed in a spectral domain optical coherence tomography scan **(A)**. Plate **(B)** demonstrates an enlarged view of the area marked by a dashed rectangle in plate **(A)**. The choriocapillaris layer appears as a hyporeflective band just next to the outer border of the retinal pigment epithelium (red arrowheads). Haller's layer (large choroidal vessels) is usually seen as reflectance-free round structures with a large diameter at the outermost parts of the choroid (white arrows). Sattler's layer (medium choroidal vessels) is located between the choriocapillaris and Haller's layer (white braces) and appears more reflective, probably because of the presence of more compact vessel walls and denser interstitial tissue.

**Figure 3:**
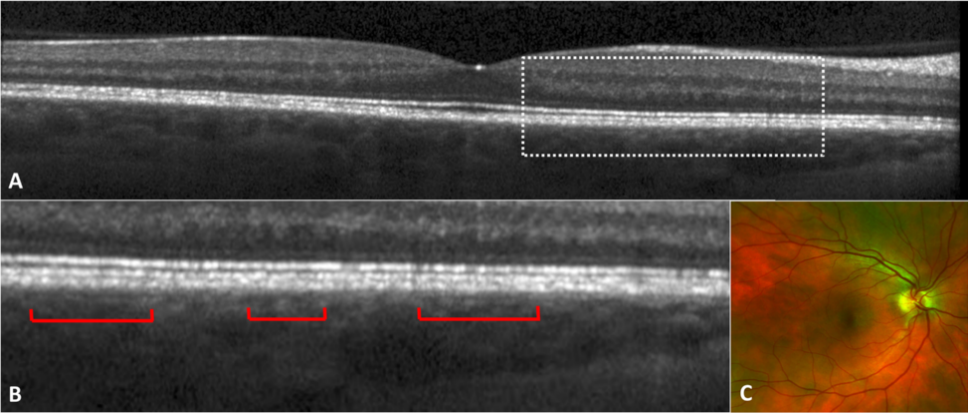
**A representative patient with convalescent stage of Vogt-Koyanagi-Harada disease.** Visual acuity was 20/20 at the time of examination. **(A)** Foveal area spectral domain optical coherence tomography scan demonstrates focal loss of the choriocapillaris layer. The area marked by a dashed rectangle is enlarged in plate **(B)** to show the areas with preserved choriocapillaris (red brackets) and those with focal loss of the choriocapillaris (between the brackets). Medium and large choroidal vessels are preserved. **(C)** Wide-field color fundus picture from the same patient.

**Figure 4:**
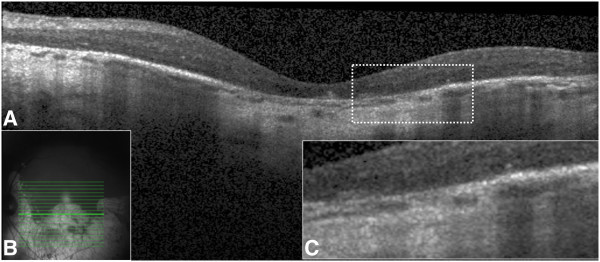
**Extensive choriocapillaris loss in a patient with long-standing convalescent stage of Vogt-Koyanagi-Harada disease with 20/100 vision.** Fundus examination demonstrates a prominent sunset glow fundus appearance with large patches of choroidal and retinal pigment epithelium atrophy **(B)**. Spectral domain optical coherence tomography scan showed extensive loss of the choriocapillaris and thinning of the retina **(A)**. The retinal pigment epithelium is atrophic demonstrating significant backscattering from the choroid and sclera **(A)**. The dashed area is magnified in plate **(C)** to show the details.

The three histologically defined layers of choroidal vasculature, the choriocapillaris, medium choroidal vessels (Sattler's layer), and large choroidal vessels (Haller's layer), were individually evaluated for the extent of preservation or loss using a single horizontal SD-OCT scan passing through the fovea (Figure 2) [10,12,13,23]. Choroidal vessel diameters were measured with the caliper tool within 3D-OCTOR software. A hyporeflective band approximately 30 μm thick, located immediately adjacent to the outer border of the RPE/Bruch's membrane complex, was considered equivalent to the choriocapillaris layer (Figure 1) [10,11]. Even in normal individuals, some focal areas of discontinuity are typically seen in this hyporeflective band because of the sinusoidal and lobular nature of this vascular network, with intervening intersinusoidal walls and communications with draining and feeding larger choroidal vessels. Sattler's layer was defined based on the presence of hyperreflective vessel walls and hyporeflective lumens of 30- to 80-μm diameters located external to the choriocapillaris layer (Figure 1) [10,11]. Larger, outer choroidal vessels of Haller's layer appeared as even larger round structures with hyperreflective walls and hyporeflective lumens with diameters greater than 80 μm (Figure 2) [10,11]. The length of the absent and preserved parts of each layer was measured using digital calipers within the 3D-OCTOR grading software and expressed as the percentage of the total length of the scan. Because areas of discontinuity in the choriocapillaris layer are expected in the normal choroid for the reasons described above, we conservatively did not deem definite loss to be present unless at least 25% of the band was absent. This choice of 25% was based on previous inspection of normal controls in prior studies (unpublished), where the hyporeflective choriocapillaris band was discontinuous for up to 25% of the scan length in some eyes. For categorical classification of the status of the choriocapillaris, the presence of the band for >75% of the horizontal foveal-centered B-scan was deemed ‘intact,’ 74% to 50% was defined as ‘mild loss,’ 49% to 25% as ‘moderate loss, and <25% as ‘severe loss’ (Figure 3). For simplicity and consistency, the same categorical system and definitions were used to grade medium choroidal vessel loss. Since large choroidal layer vessels are not continuously or evenly distributed across the entire length of an SD-OCT B-scan of the normal eye [10,11], any visible large choroidal vessel was graded as evidence of the presence of Haller's layer, and the absence of these vessels in the horizontal foveal scan was considered to be evidence of loss of Haller's layer (Figure 4).

Structural changes of the retina and RPE, including cystoid macular edema, full-thickness and lamellar macular hole, external limiting membrane (ELM) disruption, ellipsoid zone (EZ) disruption, RPE hyperplasia/hypertrophy, presence of an epiretinal membrane (ERM), and evidence of RPE atrophy, were also graded on the sub-foveal SD-OCT scans. The extent of ELM and EZ disruptions and RPE atrophy was graded based on a grading scheme of 0 to 3, with grade 0 defined as no disruption/atrophy, grade 1 defined as loss of 1% to 33% of the band, grade 2 defined as 34% to 66% loss, and grade 3 indicating disruption or loss of these bands in more than 66% of the length of the horizontal sub-foveal SD-OCT scan. Any disruption/atrophy within the central 1,000 μm of the scan (foveal center area) was considered grade 3.

Snellen visual acuity (VA) was converted to the logarithm of the minimal angle of resolution (logMAR) and presented as mean ± standard deviation (SD). Continuous data were compared using Student's *t* test. The preservation or disruption of three layers of choroidal vessels in uveitis and control eyes was compared using the chi-square test. The association of choroidal thickness and volume data with VA was analyzed with Spearman correlation analysis. Choriocapillaris loss grading and SD-OCT features and VA were correlated using Pearson correlation coefficients. The accepted level of significance was considered to be *P* < 0.05.

## Results

Twenty-two eyes of 13 patients (four males and nine females) with convalescent stage of VKH [19] and 17 eyes of 9 normal controls (three males and six females) were included. Mean ± SD age of the patient group (43 ± 11 years, range 26 to 65 years) did not differ significantly from that of the controls (35.6 ± 3.7 years, range 32 to 40 years, *P* = 0.07). Mean spherical equivalent of the patients with available refraction data (8 of 22) was -0.39 D, and that of the controls (all eyes had refraction data) was -2.10 D (*P* = 0.07). Visual acuity in control eyes was better than visual acuity in the eyes with VKH (mean LogMAR VA 0.17 ± 0.23, range 0 to 0.7, *P* < 0.0001). Mean VKH duration was 110 ± 109 months (median 69 months, range 9 to 360 months). In four patients, the OCT image obtained for one eye only was included. The fellow eyes in these four cases were excluded for three reasons: one eye was phthisical, allowing no view of the fundus; one eye had a history of pars plana vitrectomy for macular hole; and the SD-OCT image quality in two eyes was too poor to allow confident segmentation of the choroidal boundaries. One control eye was also excluded from the analysis because of poor scan quality. Of the 13 patients with VKH, six were on systemic immunomodulatory treatment at the time of data collection.

Average choroidal thickness at the fovea was 200 ±60 μm (median 202 μm, range 54 to 305 μm) in the VKH group and 288 ± 40 μm (median 286 μm, range 229 to 350 μm) in the control group (*P* < 0.0001). Choroidal thickness and volume in the foveal plus parafoveal zones and in the entire macula were also significantly less compared to normal controls (Table 1).

**Table 1:** Choroidal vasculature changes and choroidal thickness and volume values in convalescent VKH patients compared to controls

	**Patients**	**Controls**	***P *** **value**
	**No. 13 (22 eyes)**	**No. 9 (17 eyes)**	
Choroidal thickness, mean ± SD (range), μm			
Fovea	200 ± 60 (54 to 305)	288 ± 40 (229 to 350)	<0.0001 (Student's *t* test)
Parafovea	209 ± 61 (46 to 3.5)	278 ± 36 (209 to 333)	<0.0001 (Student's *t* test)
Entire macula	198 ± 58 (44 to 282)	268 ± 32 (202 to 318)	<0.0001 (Student's *t* test)
Choroidal volume, mean ± SD (range), μm			
Fovea	0.99 ± 0.31 (0.21 to 1.49)	1.49 ± 0.21 (1.17 to 1.82)	<0.000 (Student's *t* test)
Parafovea	1.28 ± 0.37 (0.29 to 1.9)	1.75 ± 0.23 (1.32 to 2.11)	<0.0001 (Student's *t* test)
Entire macula	5.33 ± 1.60 (1.01 to 7.33)	7.51 ± 0.93 (5.6 to 9.02)	<0.0001 (Student's *t* test)
Preservation of choroidal vessel layers			
Choriocapillaris-equivalent layer	11/22 (50%)	17/17 (100%)	<0.000 (chi-square)
Medium choroidal vessels	22/22 (100%)	17/17 (100%)	1
Large choroidal vessels	17/22 (77%)	17/17 (100%)	<0.000 (chi-square)

Choroidal thickness and volume values in the eyes with convalescent Vogt-Koyanagi-Harada disease were significantly lower than those in controls. Also, the choriocapillaris and large choroidal vessels were significantly lost in convalescent Vogt-Koyanagi-Harada disease.

**Figure 5:**
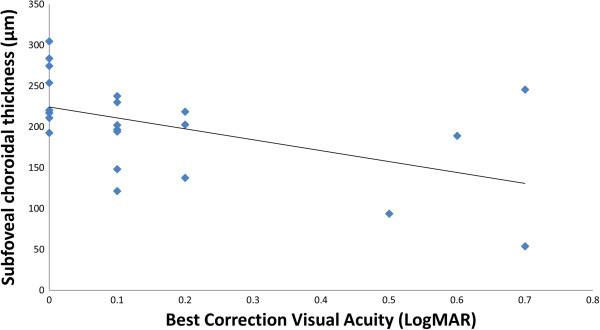
**Sub-foveal choroidal thickness in correlation to the visual acuity (*r*** **= -0.5089, *P*** **= 0.005).**

Sub-foveal choroidal thickness negatively correlated with VA (*r* = -0.5089, *P* = 0.005, Figure 5). Choroidal thickness in the parafoveal area and in the entire macula also negatively correlated with VA (*r* = -0.5358, *P* = 0.003 and *r* = -0.5413, *P* = 0.003, respectively). Choroidal thickness and volume in the fovea, the parafovea, and the entire macula did not correlate with the duration of disease. Longer disease duration, however, did show a trend for association with lower VA, albeit not statistically significant (*r* = 0.2869, *P* = 0.07).

Choroidal structures representing the choriocapillaris, medium choroidal vessels, and large choroidal vessels were graded to be intact in all controls. In patients with convalescent VKH, 11 eyes (50%) were found to have an intact choriocapillaris (i.e., preservation of the choriocapillaris-equivalent hyporeflective band in more than 75% of the length of the horizontal foveal SD-OCT scan). Two eyes had mild loss (26% to 50% loss), four eyes had moderate loss (51% to 75% loss), and five eyes had severe loss of the choriocapillaris (>75% loss) (Figure 4). The extent of choriocapillaris loss correlated with VA (Pearson correlation, *r* = 0.6868, *P* < 0.00001) but not with disease duration (Pearson correlation, *r* = 0.0324, *P* = 0.88). Medium choroidal vessels were present in all eyes; but the larger choroidal vessels could not be identified in five eyes (22.7%). These five eyes also had the most severe damage (complete loss) to the choriocapillaris layer (Figure 4). The severity of choriocapillaris loss correlated inversely with choroidal thickness at the fovea (Pearson correlation analysis, *r* = -0.4445, *P* = 0.04).

The presence of significant (grade 3) photoreceptor EZ disruption (two eyes), ELM disruption (three eyes), and RPE atrophy (two eyes) in horizontal foveal-centered SD-OCT scans correlated with lower VA (Spearman correlation coefficients = 0.65588, 0.77638, 0.65891, respectively, *P* < 0.001). The presence of ERM at the fovea also correlated with lower VA (Spearman correlation coefficient = 0.41177, *P* = 0.02). The sub-foveal choroid was significantly thinner in the eyes with retinal/RPE changes (170 ± 62 μm, range 54 to 245 μm) compared to the eyes with normal retina and RPE at the fovea (222 ± 51 μm, range 121 to 305 μm, *P* = 0.05). The choice of medications used for patients was not associated with VA or any other study variable.

**Figure 6:**
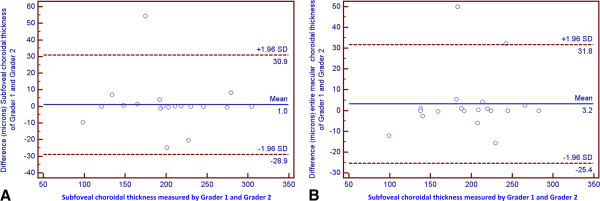
Bland-Altman plots demonstrating the interobserver agreement of sub-foveal (A) and entire macular (B) choroidal thickness measurements.

Bland-Altman plots of the mean choroidal thickness in various ETDRS subfields showed a significant agreement for the measurements by two independent graders (Figure 6). The interclass correlation coefficient for sub-foveal and entire macular choroidal thickness measurements was 0.9801 (95% confidence interval (CI) 0.9483 to 0.9923) and 0.9767 (95% CI 0.9406 to 0.9909), respectively.

## Discussion

In the current study, quantitative mapping of choroidal thickness and volume and qualitative analysis of choroidal vascular integrity showed loss of choroidal tissue including the choriocapillaris layer in the convalescent stage of VKH. Additionally, loss of the choriocapillaris and the thinning of the choroid in the sub-macula area correlated with increased duration of the disease, lower VA, and the presence of structural changes in the retina.

Normal sub-foveal choroidal thickness measured by SD-OCT ranges from 260 to 287 μm and varies by age, refractive error, and ethnicity [24-26]. Choroidal thickness varies based on the nature of posterior segment pathology as well. For instance, choroidal thickness increases in hyperopia [24], acute VKH [14,15,18], central serous chorioretinopathy [27,28], and polypoid choroidal vasculopathy [29,30]. On the other hand, loss of choroidal mass has been seen in high myopia [24], age-related macular degeneration [29], macular hole [30], degenerative choroidal disease [31], and ocular inflammation [32]. Decreased choroidal thickness and volume in chronic VKH is in contrast to what is observed in the acute phase of the disease [14,18,33]. Similarly, Nakai et al. have recently shown that sub-foveal choroidal thickness in Japanese patients with chronic VKH is significantly thinner than that in normal controls [18]. But Nakai and colleagues measured foveal choroidal thickness and did not map choroidal thickness in the entire posterior pole, as in our study [18]. We agree with Nakai et al. that choroidal thinning is most likely due to inflammatory damage to the choroidal stroma and vasculature, with the eventual resultant atrophy and fibrosis; however, this can be verified if OCT scans are correlated to the findings of simultaneously acquired indocyanine green angiography. Seeing decreased hyperreflective foci in enhanced-depth OCT images of the patients with chronic VKH, Fong et al. speculated that stromal scarring and the resulting shrinkage and dropout of small vessels in the choroid are the reasons for choroidal thinning in the convalescent stage of VKH [14]. Our observations on the loss of the choriocapillaris layer in the convalescent phase of VKH would appear to be consistent with the hypothesis of Fong et al. about the mechanism of choroidal thinning in chronic VKH [14].

Choroidal vessels are typically classified into three layers based on their diameter and permeability characteristics [26]. The choriocapillaris is particularly notable for supporting the physiologic needs of photoreceptors. Morphometric analysis of the choriocapillaris in histologic sections shows that this structure typically has a diameter of 6.5 to 9.8 μm [34]. SD-OCT studies estimate the choriocapillaris thickness to be somewhat thicker. There are several potential reasons for this apparent discrepancy. First, assessing the thickness of dynamic vascular structures by histology may be inaccurate. These vascular structures may collapse *postmortem*, leading to an underestimate compared to *in vivo* thickness. Second, optical imaging methods provide only an indirect representation of a particular structure. We know that the hyporeflective band external to the RPE/Bruch's membrane complex is in the vicinity of the choriocapillaris; however, the presence of this band on the scan is due to its optical properties, and this may not perfectly match the physical structure of the tissues or interfaces of interest. Third, the thickness of the choriocapillaris may be at the resolution limit of our SD-OCT devices. This may limit the ability to more precisely measure the true thickness of these structures. Despite these limitations, given the crucial role of the choriocapillaris in providing nourishment to the photoreceptors and the RPE and the involvement of the choroid by many ocular inflammatory diseases, evaluation of the choroid may be useful for predicting visual outcomes in eyes with uveitis. In our series, the choriocapillaris layer appeared to show varying degrees of loss in 50% of the eyes with VKH during the convalescent stage. Additionally, the extent of choriocapillaris loss correlated with lower VA (Pearson correlation, *r* = 0.6868, *P* < 0.00001). In eyes with complete loss of the choriocapillaris layer, the outer retina was also atrophic, resulting in lower VA. These findings would appear consistent with histopathologic reports from advanced and long-standing cases of chronic VKH [6]. Thus, evaluating the status of the choriocapillaris with foveal SD-OCT scans in eyes with VKH may be useful for gauging the functional potential of the eye. Choroidal thickness and volume appear to be reduced in long-standing cases of VKH, but these measurements cannot always be performed in the clinic. Assessment of the integrity of the choriocapillaris layer may prove to be an easier and more practical clinical tool for predicting the visual prognosis of patients with VKH during the convalescent stage.

Choroidal thinning and loss of the choriocapillaris were associated with retinal structural changes in the fovea and lower VA. Outer retinal damage is frequently seen in long-standing uveitis, including VKH [26,31]. In our series of VKH patients at the convalescent stage, significant photoreceptor EZ and ELM disruptions, ERM, and severe RPE alteration were observed in a small proportion of patients (9% to 14% of eyes); however, the presence of such retinal and RPE structural abnormalities correlated with lower VA and thinner choroid. Inflammatory damage is considered the underlying cause for loss of the choroidal vasculature and outer retinal damage in the setting of chronic VKH. Decreased choriocapillaris density in the fovea may compromise the overlying retina and visual function. Choriocapillaris dropout is proposed to play a major role in the pathogenesis of photoreceptor damage in age-related macular degeneration. In a mouse model of progressive choriocapillaris loss, choriocapillaris atrophy was accompanied by extensive retinal atrophy and outer retinal loss [35]. A similar process may play a role in retinal damage and decreased vision in long-standing VKH cases with damage to the choriocapillaris. Further longitudinal morphometric analysis of the choroidal structure may help clarify the process leading to choroidal thinning in long-standing inflammatory choroiditis.

Our study has several limitations which must be considered when assessing the results. First, it is a retrospective review and thus potentially subject to confounders such as selection bias. In addition, the population of patients was non-homogenous with patients treated by various agents with potentially varying degrees of success. Second, the overall sample size is small, thus reducing the power to detect smaller differences between the various groups/features under study. However, assembling a large cohort of patients with chronic VKH is a difficult task. Third, although we made an effort to match our cases with controls of a similar younger age, the control group was still slightly younger (not statistically significant), and the choroid is generally thicker in younger patients. On the other hand, the control group was slightly more myopic, and the choroid is generally thinner in myopes. Overall, we believe that these two potential imbalances may have canceled one another out. Fourth, our grading definitions (such as the percentage of discontinuity of the layer) were somewhat arbitrary. On the other hand, excellent grading reproducibility was achieved using these definitions. However, correlating SD-OCT images to findings of indocyanine green angiography could provide a better overall evaluation of the choroid in uveitis. Despite the limitations, our study has several strengths, including the use of trained experienced graders and a standardized grading protocol with documented reproducibility. Also, in VKH, future studies during the long course of disease should include linear evaluation of the choroidal stroma and choroidal vascular changes.

## Conclusions

In summary, choroidal atrophy takes place in eyes with long-standing VKH. The atrophy correlates with uveitis duration, suggesting that patients with a longer duration of disease tend to have thinner choroids. Such loss of choroidal thickness and volume appears to be, in part, due to loss of choroidal vasculature, including the choriocapillaris layer. Such changes are associated with atrophy of the overlying retina and RPE and lower visual acuity. Also, sub-foveal choroidal thickness, but not choroidal volume values, correlates with visual acuity. Thus, evaluating sub-foveal choroidal thickness and the changes in the choriocapillaris layer may be a useful approach in evaluation of visual outcome in patients with convalescent stage of VKH.

## Competing interests

SriniVas Sadda is a consultant for Carl Zeiss, Meditec, and Optos. Sadda receives research funding from Carl Zeiss, Meditec, Optovue, and Optos. Other authors declare that they have no competing interests.

## Authors' contributions

HN, NAR, and SS participated in the design of the study and coordination and helped draft the manuscript. HN, AH, ZH, and YO performed the choroidal thickness measurements. HN performed the statistical analysis. All authors read and approved the final manuscript.
